# Fatty acid and metabolomic profiling approaches differentiate heterotrophic and mixotrophic culture conditions in a microalgal food supplement 'Euglena'

**DOI:** 10.1186/s12896-016-0279-4

**Published:** 2016-06-02

**Authors:** Min Zeng, Wenlong Hao, Yongdong Zou, Mengliang Shi, Yongguang Jiang, Peng Xiao, Anping Lei, Zhangli Hu, Weiwen Zhang, Liqing Zhao, Jiangxin Wang

**Affiliations:** Shenzhen Key Laboratory of Marine Bioresource & Eco-environmental Science, Shenzhen Engineering Laboratory for Marine Algal Biotechnology, College of Life Science, Shenzhen University, Shenzhen, 518060 People’s Republic of China; Nanshan District key lab for biopolymers and safety evaluation, Shenzhen University, Shenzhen, 518060 People’s Republic of China; Laboratory of Synthetic Microbiology, School of Chemical Engineering and Technology, Tianjin University, Tianjin, 300072 People’s Republic of China; Department of Food Science and Engineering, College of Chemistry and Environmental Engineering, Shenzhen University, Shenzhen, Guangdong China

**Keywords:** *Euglena*, Metabolomics, Lipids, Fatty acid, Heterotrophic, Mixotrophic

## Abstract

**Background:**

Microalgae have been recognized as a good food source of natural biologically active ingredients. Among them, the green microalga *Euglena* is a very promising food and nutritional supplements, providing high value-added poly-unsaturated fatty acids, paramylon and proteins. Different culture conditions could affect the chemical composition and food quality of microalgal cells. However, little information is available for distinguishing the different cellular changes especially the active ingredients including poly-saturated fatty acids and other metabolites under different culture conditions, such as light and dark.

**Results:**

In this study, together with fatty acid profiling, we applied a gas chromatography–mass spectrometry (GC-MS)-based metabolomics to differentiate hetrotrophic and mixotrophic culture conditions.

**Conclusions:**

This study suggests metabolomics can shed light on understanding metabolomic changes under different culture conditions and provides a theoretical basis for industrial applications of microalgae, as food with better high-quality active ingredients.

## Background

In recent years, it was found that microalgae are a good source of natural active ingredients [1], and its chemical composition shows a great deal of diversity. Since environmental factors such as temperature, salinity, light, nutrition etc. could affect the chemical composition of microalgal cells, changes in environmental parameters can stimulate or inhibit the biosynthesis of a natural sources of biologically active ingredient [2]. A variety of natural sources of biologically active ingredients from microalgae include carotenoids, phycobilin, fatty acids, polysaccharides, vitamins and sterols [3].

Among them, *Euglena* is a very promising food and nutritional supplement. This photosynthetic green protozoan contains no cell wall, so that nutrients inside the cells have a high availability to consumers. It is not only rich in essential poly-unsaturated fatty acids (PUFAs) [4], proteins [5] and antioxidants such as β-carotene, vitamins C [6] and E [7, 8], but also accumulates a large amount of an unique Euglenoid starch-like product paramylon. As a β-1,3-glucan, paramylon can boost the immune system [9], and sulfated paramylon is also resistant to the effect of HIV [10]. After incorporated into the diet, paramylon can also reduce cholesterol in the blood [11]. Most Euglenoid species have chloroplasts containing chlorophyll a and b and can grow auto-trophic. Some species, such as genus of *Euglena* (*Euglena gracilis*), can also bloom in the dark with rich organic water. However, little information is available for distinguishing the different reaction and changes under different culture conditions, such as light and dark.

Compared to transcriptomics and proteomics, metabolomics studies at the cellular level have many advantages [12], such as both transcriptomics and proteomics need the complete genome sequences or reference genomes, while metabolomics does not need, and biological pathways are similar, thus metabolomics analyses could be shared across species. With the continuous development of innovative detection techniques and data processing methods, metabolomics research scope has been involved in many fields, such as botany [13, 14], food and nutrition sciences [15, 16], toxicology studies [17], clinical diagnosis [18] and more, demonstrating that metabolomics could be a powerful complementation to other “omics” approaches in fully deciphering the metabolic networks.

In this study, we applied a gas chromatography–mass spectrometry (GC-MS) polar metabolite screen as well as fatty acid profiling to analyze cellular responses and changes of *E. gracilis* under different culture conditions. Significantly different fatty acid compositions were unraveled, and the metabolomics also distinguished the groups under different culture conditions. This study provides a proof-of-concept in how metabolomics can be applied to profile changes and provide a theoretical basis for industrial applications of microalgae as food supplements with high-valued biological active ingredients.

## Methods

### Microalgal strains and culture conditions

*Euglena gracilis* CCAP 1224/5Z was purchased from CCAP (Culture Collection of Algae and Protozoa) and maintained in our lab at Shenzhen University. This strain is the same as SAG 1224–5/25, UTEX 753, IAM E-6, ATCC 12894 and UTCC 95 in the other culture centers all over the world. The modified heterotrophic acid culture medium [19] was used for *E. gracilis*. Log phase microalgal cells were inoculated into 250 mL Erlenmeyer flasks containing 50 mL fresh medium, at 23 ± 1 °C with continuous light of 50 μmol/m^2^ s light density as the mixotrophic culture, and aluminum foil was used to wrap the flasks for the dark treatment as the heterotrophic culture. Growth curves and cell numbers were determined by absorbance 750 nm and the blood cell counting chamber under normal light microscopy, respectively.

### Fatty acid composition and quantification

Total fatty acid extraction and fatty profiling were performed and determined as described by Bligh [20] and Lu [21] with slight modifications, which provided comparative information of the lipid productivities of different cultivation conditions [22], especially used in several microalgae-related researches for lipid extraction [23, 24].

### Euglenoid paramylon extraction and quantification

Paramylon extraction and determination was conducted as previously reported [25], total 5 mg frozen dried cell pellets was used as the input. Followed by acetone enrichment and SDS precipation, paramylon was dried and dissovlved in 0.5 M NaOH. The contents of paramylon were then determined using glucose as the standard.

### GC-MS-based metabolomic analysis

For metabolomic analysis, ~10^8^ cells were collected by centrifugation at 8,000 × g for 10 min at 4 °C (Eppendorf 5430R, Hamburg, Germany) from cultures of the *E. gracilis* on day 4, 7, and 9, respectively. The cell pellets were immediately frozen in liquid nitrogen and then stored at −80 °C before use. The metabolomic analysis was conducted as described previously [26, 27]. All chemicals used for metabolome isolation and GC-MS analyses were obtained from Sigma-Aldrich (Taufkirchen, Germany). For metabolomic analysis, cells were collected from samples at day 4, 7, and 9., respectively. The metabolomic analysis protocol included the following. *i*) *Metabolome extraction*: Cells were resuspended in 1 mL of cold 10:3:1 (v/v/v) methanol/chloroform/H_2_O solution (MCW), frozen in liquid nitrogen, and thawed five times. Supernatants were collected by centrifugation at 14,000 *g* for 3 min at 4 °C (Eppendorf 5430R, Hamburg, Germany). To normalize variations across samples, an internal standard (IS) solution (100 μg/mL U-13C-sorbitol,10 μL) was added to 100 μL of supernatant in a 1.5 mL microtube before it was dried by vacuum centrifugation for 2–3 h (4 °C); *ii*) *Sample derivatization*: the samples were dissolved in 10 μL of methoxyamine hydrochloride (40 mg/mL in pyridine), shaken at 30 °C for 90 min, added to 90 μL of *N*-methyl-*N*-(trimethylsilyl)trifluoroacetamide (MSTFA), and incubated at 37 °C for 30 min to trimethylsilylate the polar functional groups. The derivate samples were collected by centrifugation at 14,000 *g* for 3 min, and the supernatant was used directly for GC/MS analysis; *iii*) *GC-MS analysis*: Analysis was performed on a GC-MS system-GC 7890 coupled to an MSD 5975 (Agilent Technologies, Inc., Santa Clara, CA) equipped with a HP-5MS capillary column (30 m × 250 mm id), with 70 eV of electron impact ionization. Two microliters of derivatized sample was injected in splitless mode at 230 °C injector temperature. The GC was operated at a constant flow of 1 mL/min helium. The temperature program started isocratic at 45 °C for 2 min, followed by temperature ramping of 5 °C/min to a final temperature of 280 °C, and then held constant for additional 2 min. The range of mass scan was *m*/*z* 38–650; *iv*) *Data processing and statistical analysis*: The mass fragmentation spectrum was analyzed using the automated mass spectral deconvolution and identification system (AMDIS) to identify the compounds by matching the data with the Fiehn Library and the mass spectral library of the National Institute of Standards and Technology (NIST). Peak areas of all identified metabolites were normalized against the internal standard and the acquired relative abundances for each identified metabolite were used for future data analysis [26, 27].

### Principal component analysis (PCA) analysis of metabolomics

All metabolomics profile data was first normalized by the internal control and the cell numbers of the samples, after removing all singlets, i.e. masses detected only in one sample out of the eighteen analyzed, and then subjected to principal component analysis using software SIMCA-P 11.5.

### Statistical analyses

Significant dissimilarity between groups clustered by PCA were assessed by analysis of molecular variance (ANOVA) based on Euclidean distance using R. The PCA plot obtained for this dataset was characterized by a stress value less than 0.05, therefore, it could be considered a good representation of the distance matrix as well.

## Results and discussion

### Growths under different culture conditions

**Fig. 1:**
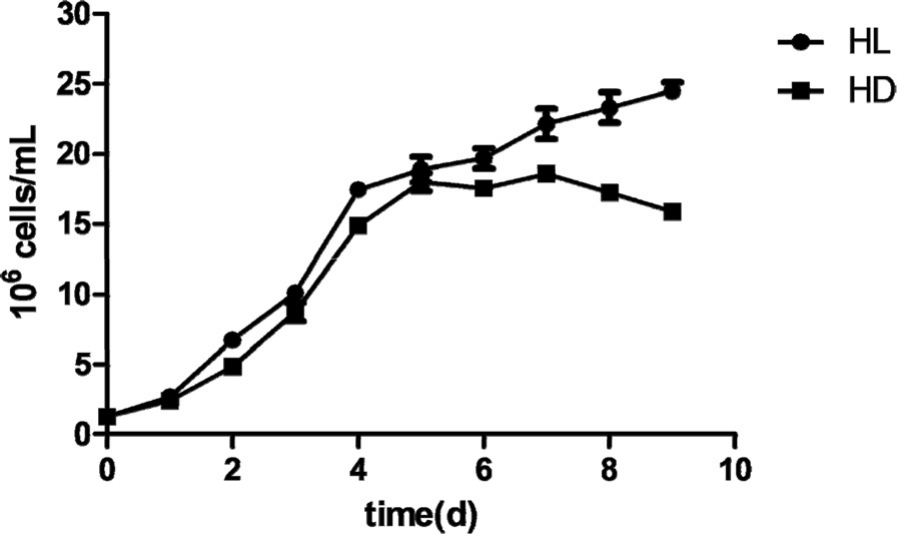
Growth curves of *E. gracilis* under mixotrophic (HL) and heterotrophic (HD) conditions

The differential growth of *E. gracilis* under mixo- (HL) and heterotrophic (HD) culture conditions were shown in the Fig. 1. From inoculation to day 6, the fast growing was observed in cultures under both light and dark, with slightly more cells under light condition. It looked like that light had little effect on the growth of *E. gracilis* during this period. However, from day 7 growth starts to differ from the two conditions: microalgal cells continue to multiply at a lower rate under light, while cells under dark set a downward trend. We speculate that nutrients in the medium were adequate initially to both cells under different culture conditions, thus cells under both conditions were able to vegetative grow and reproduce. Due to the contribution of photosynthesis, so that the growth rate under light is slightly higher than cells under dark. During the period of nutrient depletion, photosynthesis can still support the growth in the light condition, while under dark cell numbers begin to decline due to inadequate nutrition. During culture, cell numbers reached the maximum 2.45 × 10^7^ cells/mL and 10.80 g dw/L (dried weight/L) biomass, and it could be possible to obtain higher cell numbers with longer culture time under light. For the cells under dark conditions, cell numbers reached highest at day 7 with 1.86 × 10^7^ cells/mL (biomass was 6.61 g dw/L), with only about 75.9 and 61.2 % of cells under light.

### Fatty acid profiling

**Fig. 2:**
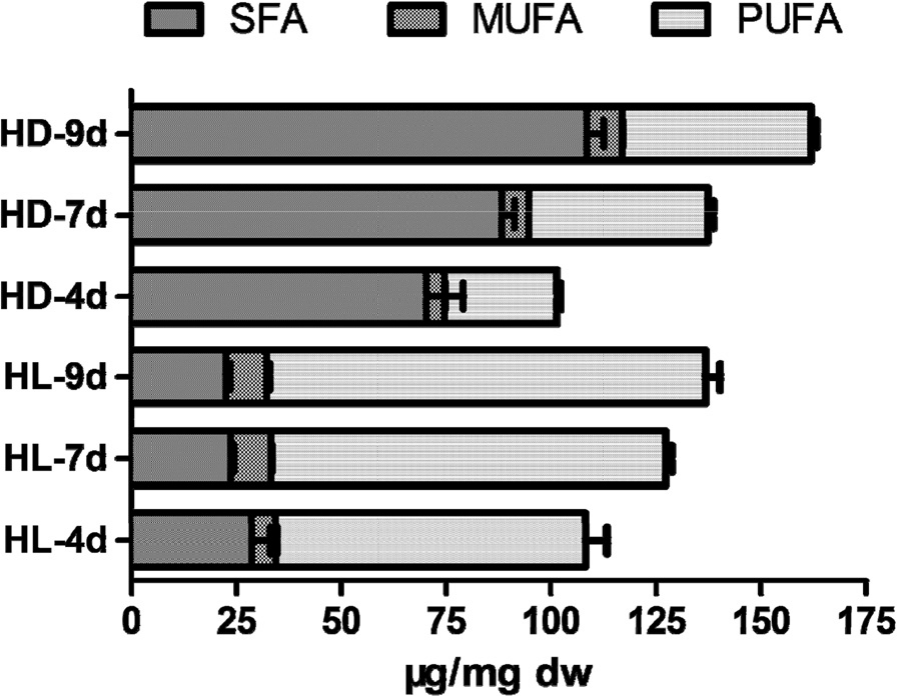
Detailed fatty acid compostions and contents under different conditions and at different time points. HL-4d, mixotrophic at day 4; HD-4d, heterotrophic at day 4

This study investigated the fatty acid composition and contents of *E. gracilis* cultured with continuous light and darkness, respectively, at different time points (day 4, 7, 9). In total, 25 kinds of fatty acids were detected, with carbon chain lengths ranging from 12–22, including 8 saturated fatty acids, and 17 unsaturated fatty acids as shown in Fig. 2 and Table 1.

**Table 1:** Fatty acid compostions (μg/mg dw)

	HL-4d	HL-7d	HL-9d	HD-4d	HD-7d	HD-9d
C12:0	1.54 ± 0.25^c^	0.84 ± 0.05^d^	0.78 ± 0.01^d^	7.29 ± 0.48^a^	4.56 ± 0.03^b^	4.16 ± 0.17^b^
C13:0	1.20 ± 0.37^c^	0.71 ± 0.06^c^	0.56 ± 0.04^c^	7.11 ± 0.75^b^	8.10 ± 0.07^a^	7.73 ± 0.26^ab^
C14:0	13.03 ± 2.93^d^	8.04 ± 0.40^d^	7.49 ± 0.32^d^	43.18 ± 6.72^c^	54.15 ± 3.11^b^	66.11 ± 2.53^a^
C15:0	1.17 ± 0.21^d^	1.21 ± 0.06^d^	1.06 ± 0.03^d^	2.65 ± 0.19^c^	5.20 ± 0.07^b^	5.62 ± 0.20^a^
C16:0	10.66 ± 0.63^c^	11.84 ± 0.14^c^	11.66 ± 0.62^c^	8.90 ± 0.52^d^	14.37 ± 0.20^b^	21.65 ± 0.92^a^
C16:1	2.89 ± 0.23^c^	5.06 ± 0.27^a^	5.45 ± 0.48^a^	2.28 ± 0.20^d^	3.32 ± 0.10^c^	4.58 ± 0.03^b^
C17:0	0.30 ± 0.07^d^	0.33 ± 0.02^d^	0.36 ± 0.03^d^	0.56 ± 0.04^c^	1.00 ± 0.04^b^	1.73 ± 0.04^a^
C16:2	3.79 ± 0.19^c^	7.58 ± 0.07^b^	10.56 ± 0.26^a^	0.53 ± 0.03^e^	0.87 ± 0.02^d^	0.78 ± 0.07^d^
C17:1	0.63 ± 0.12^b^	1.07 ± 0.13^a^	1.11 ± 0.17^a^	0.72 ± 0.09^b^	1.06 ± 0.04^a^	1.22 ± 0.06^a^
C16:3	10.96 ± 0.70^c^	14.66 ± 0.59^b^	16.05 ± 0.55^a^	1.62 ± 0.07^e^	2.67 ± 0.09^d^	2.05 ± 0.17^de^
C18:0	0.26 ± 0.06^cd^	0.24 ± 0.03^cd^	0.22 ± 0.00^d^	0.31 ± 0.03^c^	0.47 ± 0.02^c^	0.85 ± 0.03^a^
C16:4	6.06 ± 1.11^b^	7.53 ± 0.59^a^	8.01 ± 0.88^a^	0.12 ± 0.02^c^	0.17 ± 0.01^c^	0.18 ± 0.01^c^
C18:1	2.14 ± 0.12^d^	3.39 ± 0.05^a^	3.14 ± 0.17^b^	1.29 ± 0.04^f^	1.91 ± 0.02^e^	2.53 ± 0.14^c^
C18:2ω6	6.00 ± 0.33^c^	9.98 ± 0.12^b^	12.74 ± 0.39^a^	1.14 ± 0.06^e^	1.71 ± 0.06^d^	1.72 ± 0.05^d^
C18:3ω3	19.40 ± 1.64^b^	23.54 ± 0.63^a^	25.03 ± 1.44^a^	1.71 ± 0.06^c^	2.51 ± 0.11^c^	1.88 ± 0.01^c^
C20:2ω6	2.50 ± 0.20^c^	2.72 ± 0.23^c^	2.74 ± 0.14^c^	2.66 ± 0.12^c^	5.06 ± 0.19^b^	5.76 ± 0.16^a^
C20:3ω6	1.47 ± 0.11^c^	1.09 ± 0.04^d^	0.90 ± 0.04^e^	1.41 ± 0.05^c^	1.95 ± 0.08^a^	1.67 ± 0.05^b^
C20:4ω6	4.02 ± 0.33^d^	5.05 ± 0.25^c^	5.22 ± 0.26^c^	3.86 ± 0.17^d^	7.22 ± 0.25^b^	8.17 ± 0.31^a^
C20:4ω3	0.74 ± 0.05^c^	1.02 ± 0.03^b^	1.25 ± 0.09^a^	0.53 ± 0.02^e^	0.62 ± 0.03^d^	0.64 ± 0.03^d^
C20:5ω3	5.29 ± 0.25^d^	6.07 ± 0.19^c^	6.60 ± 0.28^ab^	3.81 ± 0.10^e^	6.30 ± 0.25^bc^	6.85 ± 0.14^a^
C24:0	0.46 ± 0.07^b^	0.49 ± 0.06^b^	0.42 ± 0.00^b^	0.31 ± 0.04^c^	0.43 ± 0.01^b^	0.67 ± 0.02^a^
C22:4ω6	0.46 ± 0.17^a^	0.26 ± 0.03^a^	0.17 ± 0.01^b^	0.43 ± 0.02^a^	0.47 ± 0.01^a^	0.50 ± 0.02^b^
C22:5ω6	6.66 ± 0.70^c^	8.07 ± 0.34^b^	8.67 ± 0.22^ab^	4.97 ± 0.15^d^	7.94 ± 0.26^b^	9.12 ± 0.28^a^
C22:5ω3	0.79 ± 0.28^a^	0.47 ± 0.02^bc^	0.33 ± 0.01^c^	0.57 ± 0.03^abc^	0.57 ± 0.02^abc^	0.64 ± 0.03^ab^
C22:6ω3	5.73 ± 0.53^a^	5.95 ± 0.21^a^	6.23 ± 0.26^a^	3.33 ± 0.05^c^	4.53 ± 0.22^b^	5.01 ± 0.25^b^
Sum	108.13 ± 5.87^c^	127.21 ± 2.23^b^	136.75 ± 4.59^b^	101.30 ± 9.46^c^	137.17 ± 1.50^b^	161.82 ± 5.29^a^

a, b, c, d, e, and f shows significant difference between each other

Accumulation of total fatty acids (TFAs) that were observed increased with the process of culture, under both the light and dark conditions. For instance, the TFA accumulated under light was 108.13 μg/mg dw at day 4, while the numbers increased to 127.21 and 136.75 μg/mg dw at day 7 and 9 respectilvey. Similarly, the TFA contents were 101.30, 137.17 and 161.82 μg/mg dw at day 4, 7 and 9 of dark cultures. In addition, the TFA at the late culture (day 9) in the dark was significantly more than that of the same day cultured under the light.

**Fig. 3:**
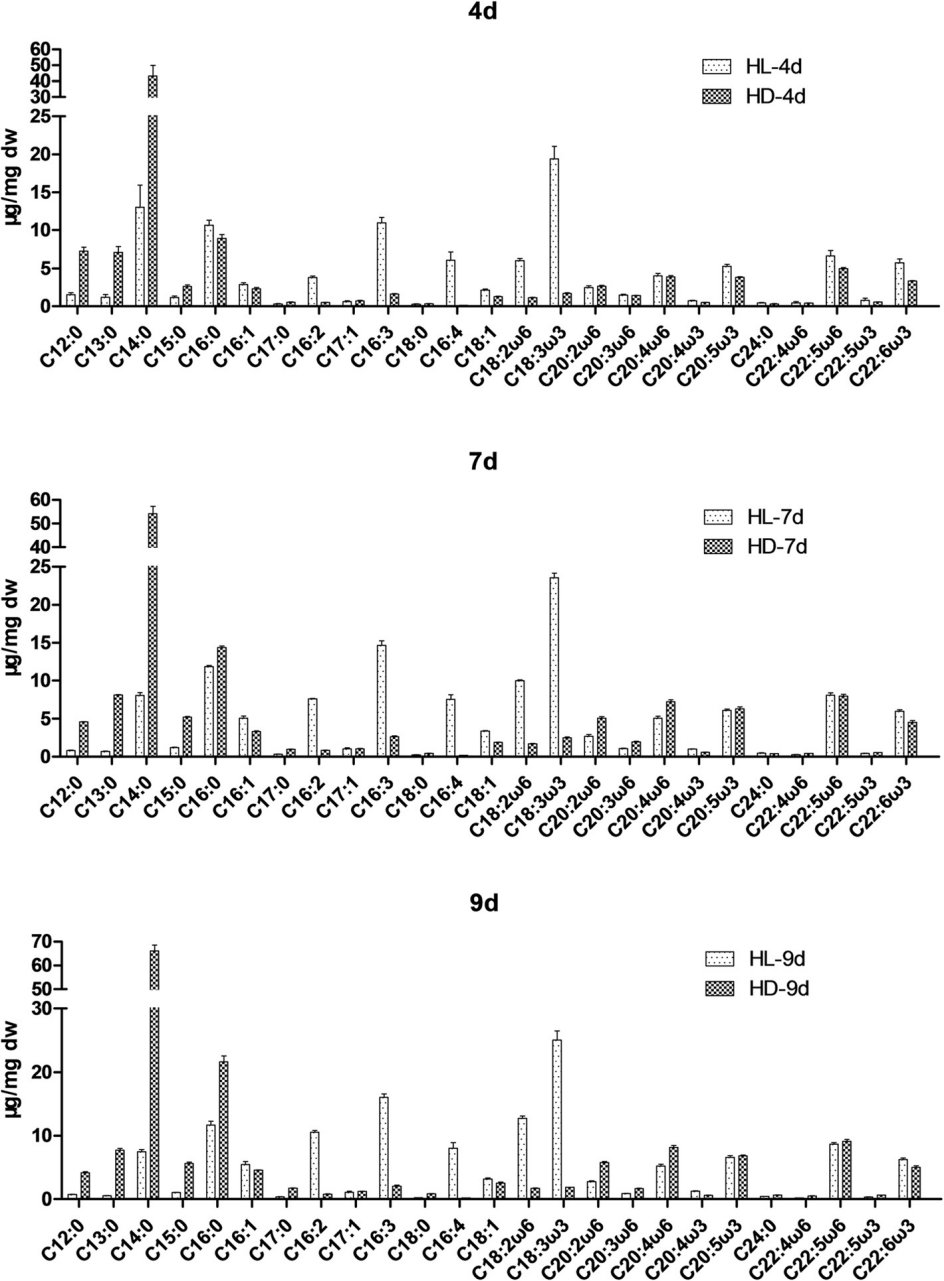
Total fatty acids, saturated fatty acids (SFA), monounsaturated saturated fatty acids (MUFA) and poly-unsaturated fatty acids (PUFA) under different culture conditions. HL-4d, mixotrophic at day 4; HD-4d, heterotrophic at day 4

Interestingly, the fatty acid types under different culture conditions also showed significant difference (Fig. 3), i.e., unsaturated fatty acids (PUFAs) were predominant under light (68.32–76.41 % of TFA), followed by saturated fatty acids (SFA) with 16.49–26.46 %, and monounsaturated saturated fatty acids (MUFA) with 5.23–7.48 %. Instead, SFAs accounted for 64.36–69.41 % under dark, followed by PUFAs with 26.35–27.79 %, and MUFAs with 4.24 –5.15 % only. As a result, at the same time point, significantly more UFAs (both MUFA and PUFA) were observed in the light cultures than under dark conditions, while for the SFA was the opposite.

In the light culture, contents of PUFAs (Table 1), C16:2, C16:3, C16:4, and C18:3ω3, were also significantly more than those under the dark. In contrast, higher levels of C12:0, C13:0, C14:0 and C16:0 FAs were observed under the dark culture, at all the culture times (day 4, 7 and 9). During the culture process, different FAs showed different trends, for instance, contents of some FAs were increased such as C18: 3ω3, C16: 3 under light and C14: 0, C16: 0 under dark; others remained unchanged, such as C14: 0, C16 : 0 under light. For the essential fatty acids EPA (C20:5ω3) and DHA (C22:6ω3), the highest content of EPA was observed under dark as 6.85 μg/mg dw, and the content of DHA seemed only connected with light, as they were significantly higher than those under dark, with no significant difference among different culture times at 5.73–6.23 μg/mg dw.

Individually, during the culture process, both ω3 and ω6 contents were significantly increased under light and dark groups (Table 2), but ω3/ω6 ratio was significantly decreased. At the same time point, contents of ω3, ω6 or ratio of ω3/ω6 under light were significantly higher than the darkness.

**Table 2:** Fatty acids classes and distribution (μg/mg dw)

	HL-4d	HL-7d	HL-9d	HD-4d	HD-7d	HD-9d
SFA	28.61 ± 4.31^d^	23.71 ± 0.73^d^	22.55 ± 1.02^d^	70.31 ± 8.71^c^	88.28 ± 3.03^b^	108.53 ± 4.02^a^
MUFA	5.65 ± 0.40^c^	9.51 ± 0.43^a^	9.70 ± 0.81^a^	4.30 ± 0.22^d^	6.29 ± 0.15^c^	8.33 ± 0.19^b^
PUFA	73.87 ± 5.06^c^	93.99 ± 1.52^b^	104.50 ± 3.52^a^	26.69 ± 0.87^e^	42.60 ± 1.50^d^	44.96 ± 1.50^d^
SFA/(MUFA + PUFA)	0.36 ± 0.07^d^	0.23 ± 0.01^d^	0.20 ± 0.00^d^	2.27 ± 0.23^a^	1.81 ± 0.12^c^	2.04 ± 0.06^b^
ω3	31.95 ± 2.26^c^	37.05 ± 0.61^b^	39.44 ± 1.85^a^	9.95 ± 0.26^e^	14.53 ± 0.59^d^	15.02 ± 0.45^d^
ω6	21.11 ± 1.40^d^	27.17 ± 0.90^b^	30.44 ± 0.96^a^	14.48 ± 0.54^e^	24.35 ± 0.82^c^	26.93 ± 0.83^b^
ω3/ω6	1.51 ± 0.03^a^	1.36 ± 0.04^b^	1.30 ± 0.05^c^	0.69 ± 0.01^d^	0.60 ± 0.00^e^	0.56 ± 0.00^e^

a, b, c, d, and e shows significant difference between each other

In summary, the light does not affect the fatty acids types in *E. gracilis*, but significantly affects the FA saturation level. In the dark, *E. gracilis* could accumulate a considerable amount of short-chain FA with the majority of C12-C16:0; In contrast, only under the light conditions, desaturase activity was enhanced to generate a set of UFAs. The observation in this study agreed with the surveys of Regnault et al. [28] that when carbon source exists *Euglenophyta* accumulated lots of C14 and C16 fatty acids.

### Paramylon quantification

**Fig. 4:**
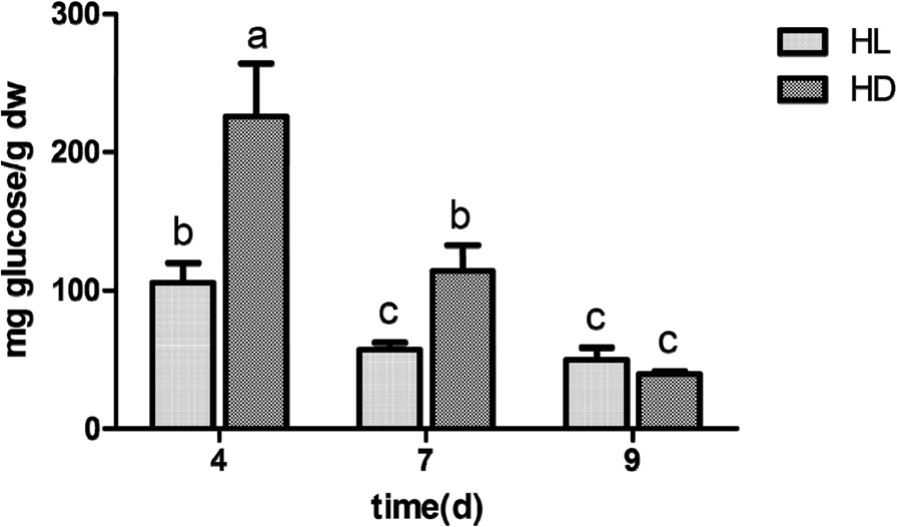
Paramylon contents under different culture conditions. a, b, and c show significant difference between each other

Paramylon, the high-valued product from *E. gracilis*, was also one of our interests. The highest paramylon contents were observed at day 4 under both conditions compared to the day 7 and 9, showing the decreasing trends in the process of culture (Fig. 4). Expectedly, higher contents of paramylon were observed under dark at days 4 and 7, while there was no significant difference at day 9.

It was reported the effect of growth conditions on paramylon contents in wild type and chloroplast-less mutant of *E. gracilis* [29]. We proposed that paramylon in cells reached a maximum at 24 h after inoculation, and the contents were then decreased with accelerated growth and reproduction, and probably degraded for the synthesis of other components used in the new cells. Compared to cells under light, cells in the dark accumulated much more paramylon at the beginning but they were consumed very quickly to the similar levels as under light.

### Metabolomic analysis

**Fig. 5:**
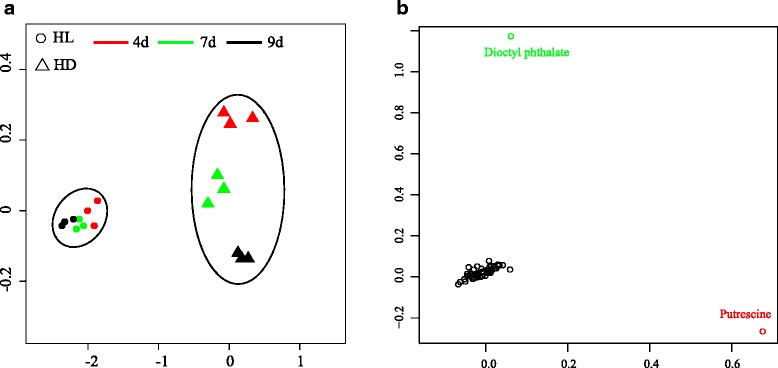
Metabolomic analysis of cells under different culture conditions. PCA plots of group clusters (**a**) and all metabolites (**b**)

Regarding the polar metabolite profiling, for metabolomics, we total obtained in total 18 sets of data, 9 for each culture condition and triplicates for each data point. Under this experiment two different culture conditions, a total of 86 metabolites were obtained. Overall, the three biological replicates of each sample was relatively distributed close to each other, and different samples under the same culture condition were separated from one another, indicating the reliability and reproducibility of metabolomics approaches in this study. Under different culture conditions, HL and HD groups could be significantly separated based on the metabolites (Fig. 5a). HD groups from different time points could be distinguished, but with a smaller difference as those between HL and HD. Surprisingly, groups under light at day 7 and 9 were assorted very closely, indicating few metablomic changes under these time points.

GC-MS based metabolomics analysis can achieve a good coverage of polar metabolites, such as amino acids and organic acids, and allow analysis of a wide range of chemical metabolite classes in a single run [30]. In the previous study, we developed an optimized protocol characterizing the time-series metabolic responses for metabolite isolation and MS analysis, and achieved identification of more than 65, 60 and 111 chemically classified metabolites from *Escherichia coli* [26], cyanobacterium *Synechocystis* sp. PCC 6803 [31], and heterotrophic dinoflagellate microalga *Crypthecodinium cohnii* [32], respectively. In this study, we followed the similar protocol with minor modification by collecting the cells under light and dark culture at day 4, 7 and 9. A good separation of intracellular metabolites was achieved on the GC column and further MS analysis allowed the chemical classification of a total of 86 metabolites from *E. gracilis*, including various fatty acids, amino acids, sugars and organic acids. Metabolites detected in *E. gracilis* are much more than those in *E. coli* and *Synechocystis*, probably due to *E. gracilis* is a eukaryote.

PCA score plots were first applied to evaluate the similarities and differences between a total of 86 metabolomic profiles (Fig. 5b). In general, the score plots of the GC-MS metabolomic profiles revealed overall good reproducibility between biological replicates and good separation between different sample clusters. Combined with previous growth data, this result suggested that members of the light cultivated groups had significantly different metabolisms. Hence GC-MS could be used to reflect cells metabolism changes caused by different culture conditions.

PCA score plots revealed that 86 detected metabolites were plotted in the intermediate position, indicating that no dramatic metabolite changes occurred in the culture throughout the process. However, the distributions of some compounds were relatively significant, which may be associated with different culture conditions. These metabolites were dioctyl phthalate and putrescine. Interestingly, there is no report about the impact of dioctyl phthalate on cellular metabolism or photosynthesis. The emergence of this metabolite is most likely an artifact from extraction/derivatization processes. Diamines and polyamines such as putrescine and spermidine are specific regulators of cellular and metabolic processes which can stimulate active transport of metabolites, and affect the functioning of enzymes and ion pumps in the cellular membranes. They also stimulate the photosynthetic process in green microalgae [33]. Thus, putrescine could be the potential target bio-stimulator for *E. gracilis* growth.

## Conclusions

In summary, different culture conditions in *E. gracilis* produced significant different profiles of fatty acids and metabolites. Compared to cells under light, cells under dark accumulated much more paramylon at the beginning of cultivation. It was also shown in this study that the light does not affect the fatty acids types in *E. gracilis*, but significantly affects the FA saturation level. In the dark, *E. gracilis* could accumulate a considerable amount of short-chain FA, but only under the light conditions, desaturase activity was enhanced to generate a set of UFAs. The GC-MS could reflect cell metabolism changes caused by different culture conditions and the potential target bio-stimulator, putrescine, for *E. gracilis* growth was also detected via metabolomic analysis in this study.

## Abbreviations

dw/L, dried weight/L; FAs, fatty acids; HD, heterotrophic; HL, mixotrophic; MUFA, monounsaturated saturated fatty acids; PCA, principal components analysis; PUFAs, poly-unsaturated fatty acids; SFA, saturated fatty acids; TFA, total fatty acid
